# Adipose stromal cells primed with hypoxia and inflammation enhance cardiomyocyte proliferation rate *in vitro* through STAT3 and Erk1/2

**DOI:** 10.1186/1479-5876-11-39

**Published:** 2013-02-13

**Authors:** Ewa Przybyt, Guido Krenning, Marja GL Brinker, Martin C Harmsen

**Affiliations:** 1 University Medical Center Groningen, Department of Pathology and Medical Biology, Cardiovascular Regenerative Medicine Research Group (CAVAREM), Department of Pathology and Medical Biology, University of Groningen, Hanzeplein 1 (EA11), Groningen, GZ 9713, The Netherlands

**Keywords:** Myocardial infarction, Hypoxia, Inflammation, ADSC, Interleukin-6, Cardiomyocyte Proliferation, STAT3, Erk1/2

## Abstract

**Background:**

Experimental clinical stem cell therapy has been used for more than a decade to alleviate the adverse aftermath of acute myocardial infarction (aMI). The post-infarcted myocardial microenvironment is characterized by cardiomyocyte death, caused by ischemia and inflammation. These conditions may negatively affect administered stem cells. As postnatal cardiomyocytes have a poor proliferation rate, while induction of proliferation seems even more rare. Thus stimulation of their proliferation rate is essential after aMI. In metaplastic disease, the pro-inflammatory cytokine interleukin-6 (IL-6) has been identified as potent mediators of the proliferation rate. We hypothesized that IL-6 could augment the proliferation rate of (slow-)dividing cardiomyocytes.

**Methods:**

To mimic the behavior of therapeutic cells in the post-infarct cardiac microenvironment, human Adipose Derived Stromal Cells (ADSC) were cultured under hypoxic (2% O_2_) and pro-inflammatory conditions (IL-1β) for 24h. Serum-free conditioned medium from ADSC primed with hypoxia and/or IL-1β was added to rat neonatal cardiomyocytes and adult cardiomyocytes (HL-1) to assess paracrine-driven changes in cardiomyocyte proliferation rate and induction of myogenic signaling pathways.

**Results:**

We demonstrate that ADSC enhance the proliferation rate of rat neonatal cardiomyocytes and adult HL-1 cardiomyocytes in a paracrine fashion. ADSC under hypoxia and inflammation *in vitro* had increased the interleukin-6 (IL-6) gene and protein expression. Similar to conditioned medium of ADSC, treatment of rat neonatal cardiomyocytes and HL-1 with recombinant IL-6 alone also stimulated their proliferation rate. This was corroborated by a strong decrease of cardiomyocyte proliferation after addition of IL-6 neutralizing antibody to conditioned medium of ADSC. The stimulatory effect of ADSC conditioned media or IL-6 was accomplished through activation of both Janus Kinase-Signal Transducer and Activator of Transcription (JAK/STAT) and Mitogen-Activated Protein (MAP) kinases (MAPK) mitogenic signaling pathways.

**Conclusion:**

ADSC are promising therapeutic cells for cardiac stem cell therapy. The inflammatory and hypoxic host post-MI microenvironment enhances the regenerative potential of ADSC to promote the proliferation rate of cardiomyocytes. This was achieved in paracrine manner, which warrants the development of ADSC conditioned medium as an “of-the-shelf” product for treatment of post-myocardial infarction complications.

## Introduction

Postnatal cardiomyocytes (CM) have a limited proliferation rate that does not suffice to replenish the CM that are massively lost after Myocardial Infarction (MI). During human life span approximately half of the cardiomyocytes are replaced [1,2]. This indicates that there is a significant level of physiological proliferation of cardiomyocytes. Thus, novel therapies that promote the proliferation of CM after acute Myocardial Infarction (aMI) may alleviate post-infarct complications such as heart failure.

Over the past decade, mesenchymal stem cells (MSC) emerged as promising candidates for cardiac therapy. Stem cells and progenitor cells from sources that vary from bone marrow to adipose tissue and the heart itself have shown to be beneficial in animal models of aMI and in clinical trials [3-5]. The current dogma is that stem cells act primarily through paracrine intervention in the damaged cardiac microenvironment *i*.*e*. through secretion of trophic factors [6,7]. The secretion profile and the fate of administrated cells change upon a host microenvironment. Current research on preconditioning BM-MSC with the hypoxic and the inflammatory factors found in post-MI microenvironment improve the cardioprotective outcome of the therapeutic cells [8-10]. Thus priming Adipose tissue-derived stem cells (ADSC) for the treatment of MI with hypoxic and inflammatory conditions might result in the improvement of cardiac function.

ADSC belong to the family of MSC and are derived from the adipose vascular stromal fraction as fibroblastic, spindle-shaped, plastic adherent cells and co-express several mesenchymal markers such as CD105, CD90, CD44, CD29 or CD73 [11,12]. *In vitro*, ADSC secrete a plethora of factors that are cytoprotective, promote angiogenesis and induce proliferation of various cell types [13-15]. Indeed, in animal models of myocardial infarction, the intramyocardial administration of ADSC improved cardiac remodeling and function [16,17]. Yet, the influence of administered stem cells on the proliferation rate of cardiomyocytes is poorly studied.

In damaged tissues, interleukin-6 (IL-6) is both cytoprotective and anti-apoptotic [18,19]. However, during the late post-MI healing phase IL-6 is upregulated in the myocardium. This chronic exposure to IL-6 activates as a compensatory hypertrophic reaction of the surrounding cardiac tissue and may contribute to cardiac fibrosis. IL-6 acts as a mitogen on several cell types, *e*.*g*. on hepatocytes during liver regeneration. Furthermore, IL-6 facilitates healing of damaged skeletal muscle through mitotic stimulation of muscle progenitor cells [20,21]. IL-6 binds to the IL-6/gp130 receptor complex and activates the associated Janus Kinase (JAK), which phosphorylates, *i*.*e*. activates STAT3 (signal transduction and activator of transcription) to p-STAT3. The p-STAT3 translocates to the nucleus and initiates transcription of its responsive genes. STAT3 activation can also occur through cross-talk between other mitogenic signaling pathways, such as the mitogen activated protein kinase (MAPK) pathway [22]. One of the trophic factors readily secreted by ADSC is IL-6. Therefore, we hypothesized that IL-6 secreted by ADSC could stimulate the rate of cardiomyocyte proliferation through JAK/STAT and MAPK-dependent pathways.

## Materials and methods

### ADSC isolation and culture

Human subcutaneous adipose tissue samples were obtained after liposuction surgery, which was donated upon informed consent of the healthy patients with BMI below 30 (Bergman Clinics, The Netherlands). Adipose tissue was stored at 4°C and processed within 24 h post surgery. Following extensive washing with PBS, the tissue was enzymatically digested with 0.1% Collagenase A, (Roche Diagnostic, Mannheim, Germany) 1:1 in PBS, containing 1% bovine serum albumine (BSA; Sigma-Aldrich, Boston, MA) at 37°C for 1 h. Digested tissue was washed with PBS, 1% BSA to remove the adipocytes and lipid content. The cell pellet was resuspended in PBS, 1% BSA and subjected to Lymphoprep (Axis-Shield PoC, Oslo, Norway) density gradient centrifugation. The cells from the interface were collected and washed with PBS, 1% BSA and resuspended in DMEM (Lonza Biowhittaker, Verviers, Belgium), 10% FBS (Thermo Scientific, Hemel Hempstead, UK), 100 U/mL penicillin, 100 mg/mL streptomycin (Gibco, Invitrogen, Carlsbad, CA) and 2 mM L-glutamine (Lonza Biowhittaker, Verviers, Belgium). Cells were seeded in culture flasks at 4x10^4^/cm^2^, expanded till Passage 3 and used for experiments. The use of liposuction material as source of ADSC was approved by of the local Ethics Committee of University Medical Centre Groningen, given the fact that it was considered the use of anonymised waste material. Yet, for every one of these anonymous donations the clients gave their consent after information.

### Cardiomyocytes isolation and culture

Rat neonatal cardiac tissues were collected and kept in a head-over-head rotator at 4°C in trypsin (Gibco, Invitrogen, Carlsbad, CA) overnight. Afterwards, the tissues were enzymatically digested with 550 U of Collagenase A, (Roche Diagnostic, Mannheim, Germany) and filtered through 70 μm cell straine into the cold FCS solution (Gibco, Invitrogen, Carlsbad, CA). The cell suspension was resuspended in DMEM (Lonza Biowhittaker, Verviers, Belgium), 10% FCS (Gibco, Invitrogen, Carlsbad, CA), 100 U/mL penicillin, 100 mg/mL streptomycin (Gibco, Invitrogen, Carlsbad, CA) and 2 mM L-glutamine (Lonza Biowhittaker, Verviers, Belgium). Fibroblasts were depleted through plastic adhesion, non-adhered cells *i*.*e*. cardiomyocytes were re-seeded at 20,000 cells/cm^2^ in fibronectin-coated flasks (25 μg/ml , Harbor Bio-products, Noordwood, MA). Animal experiments, i.e. the use of neonatal rat heart for the isolation of cardiomyocytes was approved by the local committee for animal experiments of the Amsterdam University Medical Centre. Animal experimentation was approved by the local committee for care and use of laboratory animals and performed according to strict governmental and international guidelines. The investigation conformed to Guide for the Care and Use of Laboratory Animals published by the US National Institutes of Health (NIH Publication No. 85-23, revised 1996).

HL-1 murine cardiomyocytes were a kind gift of Dr. William C. Claycomb (Department of Biochemistry and Molecular Biology, Louisiana State University Health Science Center, New Orleans, LA, USA). Cells were maintained in fibronectin-coated flasks in Claycomb expansion medium (Sigma-Aldrich, Boston, MA,) supplemented with 10% FBS (Sigma-Aldrich, Boston, MA), 0.1 mM norepinephrine (Sigma-Aldrich, Boston, MA), 100 U/mL penicillin, 100 mg/mL streptomycin (Gibco, Invitrogen, Grand Island, NY) and 2mM L-glutamine (Lonza Biowhittaker, Verviers, Belgium) and kept semi-confluent at all times.

### Experimental culture conditions

Prior to co-cultures of ADSC and rat neonatal cardiomyocytes (rnCM) the cells were labeled with the CFDA SE (green) and CM-DiI (red) respectively according to the manufacturer’s instructions (both Molecular probes, Invitrogen, Grand Island, NY). Co-cultures of ADSC and HL-1 cardiomyocytes were done after lentivirus tagging with resp. lentiviruses encoding eGFP and dTomato. Briefly, on the day of transduction, cells were plated at 1× 10^6^ cells/well in serum-free growth medium containing 5 μg/ml polybrene (Sigma-Aldrich, Boston, MA). Following overnight incubation, medium was replaced with normal growth medium containing 10% FBS. The medium of HL-1 cells was changed once per 24h while ADSC medium was replenished three times a week. At five days post-transduction, cells were FACS sorted based on expression of eGFP or dTOMATO to obtain pure cell population. To determine the influence of the ADSC density on cardiomyocyte proliferation, ADSC were treated with 10 μg/ml mitomycin-C (Sigma-Aldrich, Boston, MA) for 3h, followed by extensive washing with PBS prior the co-culture with rnCM and HL-1 cells. The ADSC cell ratios plated in co-culture conditions varied from 1:1 to 1:3 for rnCM, while keeping the rnCM at 20,000 cells/cm^2.^ The ADSC ratios in co-cultures with HL-1 cells varied from 1:1 to 1:4, while keeping the HL-1 cells at 6,000 cells/cm^2^. Simultaneously, cells were labeled with 1 μM BrdUrd-bromodeoxyuridine (Sigma-Aldrich, Boston, MA) for 6h at the end of the experiment. In order to study the effect of the post-MI microenvironment on cardiomyocytes, rnCM and HL-1 cells and ADSC were cultured at ambient oxygen tension 21% O_2_ (normoxia) or at 2% O_2,_ (hypoxia). At these oxygen tensions inflammation was mimicked by continuous treatment with TNF-α or IL-1β (both 10 ng/ml Peprotech, Rocky Hill, NJ) or none as a control for 24 and 48h respectively. ADSC conditioned medium was collected after pre-treatment according to the experimental procedures for 24h. Subsequently, followed by medium replacement without the stimuli and conditioning in 0% FBS Claycomb Medium for 24h.

### Gene transcript analysis

ADSC were seeded in 12 well plates at 10,000 cells/cm^2^ in DMEM and treated according to the experimental procedures. HL-1 cardiomyocytes were seeded in 12 well plates at 10,000 cells/cm^2^ in 5% FBS Claycomb medium, afterwards cells were incubated with 10% FBS (control) and 0% FBS Claycomb medium or 0% FBS ADSC conditioned medium for 24h and treated according to the experimental procedures. Cells were harvested at the pre-determined time points and RNA was extracted using the Rneasy Mini Kit (Qiagen Inc., Valencia, CA) for ADSC and Trizol Reagent (Life technologies, Carlsbad, CA) method for HL-1 cells according to manufacturer’s protocol. Afterwards, 1 μg of total RNA was reverse transcribed using the First Strand cDNA synthesis kit (Fermentas UAB, Vilnius, Lithuania) according to manufacturer’s instructions. The cDNA equivalent of 5 ng RNA was used for amplification in 384-well microtiter plates in a TaqMAN ABI7900HT cycler (Applied Biosystem, Foster City, CA) in a final reaction volume of 10 μl containing 5 μl SyberGreen universal PCR Master Mix (BioRad, Richmond, CA) and 6 μM primer mix (forward and reverse). 6 μM of mouse Beta-2 microglobulin and human GAPDH primer mix were used as a reference gene. Cycle threshold (C_T_) values for individual reactions were determined using ABI Prism SDS 2.2 data processing software (Applied Biosystem, Foster City, CA). To determine the differences in expression, the C_T_ values were normalized to reference gene using the ΔΔCt method, normalizing for the expression of the reference gene and related to the control treatment. All cDNA samples were amplified in duplicate. (Table 1)

**Table 1:** Primer sequence for qRT-PCR

**Mouse primer**	**Forward**	**Reverse**
IL-6	5’TTCCTCTGGTCTTCTGGAG-3’	5’-TGAGGAATGTCCACAAACTG-3’
gp80	5‘-AAACAGTGTGGGAAGCAAGT-3’	5’ATGGCTGATACCACAAGGTT-3’
gp130	5’-AGCGTTGTGGAAATAGAAGC-3’	5’-GGACTTCAGGTCATCTGGAC-3’
Cyclin D1	5’-AGCTGGTGTTTGGAAGTAGG-3’	5’-AAAAGCCTCCTGTGTGAGAC-3’
Cyclin D2	5’-TTTGCCATTTCTTTCCTCTC-3’	5’-AGTGCTTCCCTTACCTCCTT-3’
c-myc	5’-GAGGAAACGACGAGAACAGT-3’	5’-AGTGCTTCCCTTACCTCCTT-3’
Bclx	5’-CCCCTGCAATTAGCTTTCTA-3’	5’-AGGCCAAGAGAACTGAGATG-3’
B2M	5’-AGAATGGGAAGCCGAACTA-3’	5’-CCGTTCTTCAGCATTTGGAT-3’
**Human primer**	**Forward**	**Reverse**
IL-6	5’-ACTTGCCTGGTGAAAATCAT-3’	5’-CAGGAACTGGATCAGGACTT-3’
GAPDH	5’-AGCCACATCGCTCAGACAC-3’	5’-GCCCAATACGACCAAATCC-3’

### ELISA

ADSC conditioned medium was collected and filtered through 0.2 μm filter to remove any residual debris. To quantify the IL-6 production by ADSC, collected media were assessed by enzyme-linked immunosorbent assay (ELISA) (R&D Systems, Minneapolis, MN) according to manufacturer’s protocol. Absorbance values for individual reactions were determined using VersaMax™ Microplate Reader with SoftmaxPro 3.0 data processing software. To assure statistically relevant data, samples were run in triplicate from three independent donors.

### Immunoblot analysis

Confluent rnCM or HL-1 cardiomyocyte cultures were serum-starved overnight. Subsequently, 50 μM Stattic (STAT3 inhibitor) (Calbiochem, Darmstadt, Germany) or 10 μM UO126 (MEK-1 inhibitor) (Promega, Madison, WI) and solvent controls were added to HL-1 cells for 2h. Next, rnCM or HL-1 cultures were treated with ADSC conditioned medium for 30min. Protein lysates from serum depleted, confluent cultures of HL- 1 cells were prepared in RIPA buffer (Thermo Scientific, Rockford, IL,) supplemented with 1% protease inhibitor cocktail and 1% phosphatase inhibitors cocktail 2, 3 (Sigma-Aldrich, Boston, MA). Cell lysates (20 μg) were run on 10% polyacrylamide electrophoresis gel and blotted onto nitrocellulose membrane according to standard protocol. Blots were blocked in Tris-buffered saline (TBS) containing 5% BSA (Sigma-Aldrich, Boston, MA) for 1 h. Subsequently, blots were incubated in TBS-1% Tween containing 5% BSA with primary antibodies to human p-STAT3 (Y705), STAT3 (Cell Signaling Technology, Beverly, MA), p-Erk1/2 (Thr 202/Tyr 204), Erk1/2 (42/44) (Cell Signaling Technology, Beverly, MA), diluted 1:1000, overnight. Afterwards, blots were washed and incubated with alkaline phosphatase-conjugated antibodies to mouse or rabbit IgG, at the dilution 1:2000 for 1 h. NBT/BCIP (Bio-Rad, VA) was used as a substrate for detection. Densitometric analysis was performed using Totallab 120 (Nonlinear Dynamic, Newcastle upon Tyne, England).

### Immunofluorescence microscopy and image analysis

rnCM and HL-1 cardiomyocytes were seeded semi-confluent onto polystyrene 8-chamber slides (Thermo Scientific, Hemel Hempstead, UK). Subsequently, cells were serum-starved in serum-free Claycomb Medium overnight. Afterwards, samples were stimulated with 10 ng/ml IL-6 (Peprotech, Rocky Hill, NJ), conditioned media of ADSC and conditioned media of ADSC supplemented with IL-6 neutralizing antibody (1.2 μg/ml) or Mock IgG (5.33 μg/ml) as a control (all R&D Systems, Minneapolis, MN) for 24 h. As a growth control, 10% FBS Claycomb Medium was used. Simultaneously, cells were labeled with 1 μM BrdUrd (Sigma-Aldrich, Boston, MA) for last 6h. Next, cells were fixed using 2% paraformaldehyde at room temperature for 20 min. After extensive washing, cells were permeabilized with 0.5% Triton X-100 in PBS (Sigma-Aldrich, MA). Samples were treated with 0.7 M HCl and 0.05% pepsin at 37°C and post-fixed with paraformaldehyde (4% in PBS). Subsequently, samples were incubated with primary antibody sheep polyclonal biotinylated α-BrdUrd (Abcam, Cambridge, UK) diluted 1:100 in PBS with 10% goat serum (First Link Ltd, UK) overnight. Samples were washed extensively and incubated with secondary antibody Streptavidin-FITC for HL-1 cells and Streptavidin-Cy 3 for rnCM (Dako, Carpinteria, CA) diluted 1:400 in 3 μM DAPI in PBS with 10% mouse serum (Sanquin, Amsterdam, The Netherlands) for 30 minutes.

To determinate the working mechanism of cardiomyocyte proliferation, serum-free cultured HL-1 cardiomyocytes were cultured in the presence of 50 μM JAK1 inhibitor (AG490) or 50 μM STAT3 inhibitor (Stattic) (Calbiochem, Darmstadt, Germany), 10 μM RAS inhibitor (FTS) (Cayman Chemical Co., Ann Arbor, MI) or 10 μM MEK inhibitor (U0126) (Promega, Madison, WI) and according controls with DMSO for 2h. Afterwards, cells were extensively washed with PBS and cultured in 5% Claycomb medium or ADSC conditioned medium in the presence of 1μM BrdUrd for 6 h. Next, samples were fixed using 2% paraformaldehyde and proceed with BrdUrd staining as mentioned above. Stained samples were extensively washed and proceed with Tissue FAXS analysis to quantify percentage of BrdUrd positive HL-1 cardiomyocytes. Examination was performed by immunofluorescent microscopy using a Leica DMRXA microscope and Leica software (Leica Microsystems), and further quantification was performed by TissueFAXS using a Zeiss AxioObserver. Z1 microscope and TissueQuest cell analysis software (TissueGnostics).

### Statistics

All the data are presented as a means +/− SEM and were analysed by GraphPad Prism (version 5, GraphPad Software Inc.). Statistical significance was determined using one-way ANOVA with Bonferroni *post hoc* analysis. Values of p < 0.05 were considered statistically significant.

## Results

### ADSC promote the rate of cardiomyocyte proliferation in direct co-culture

**Figure 1:**
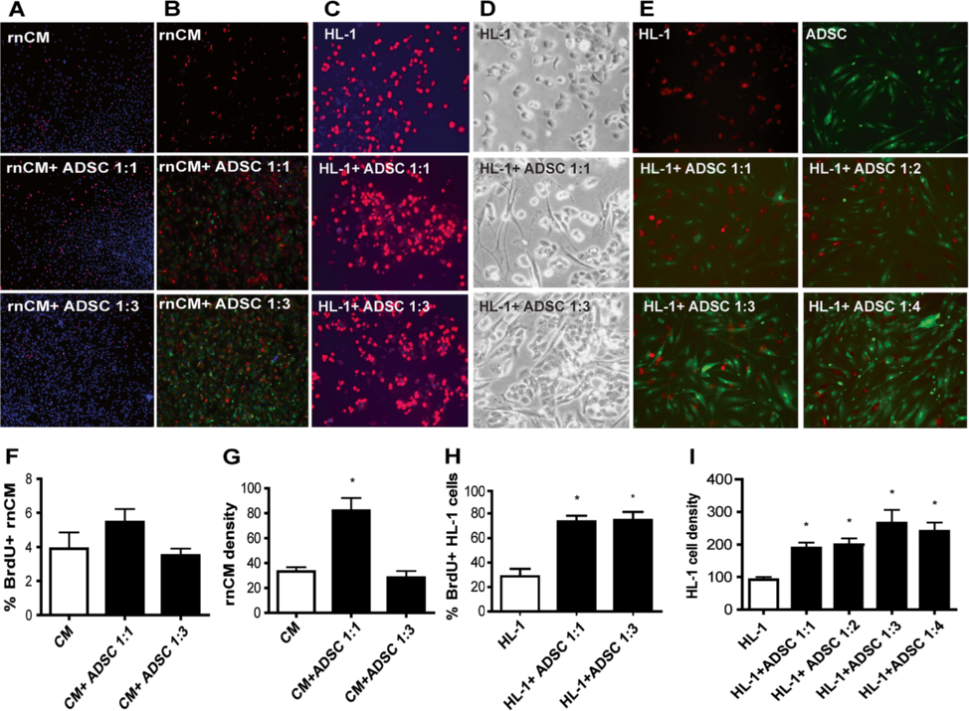
**ADSC promote cardiomyocyte proliferation rate in direct co-culture. A**) Rat neonatal cardiomyocytes and **C**) HL-1 cardiomyocytes were co-cultured with mitomycin-C treated ADSC in a 1:1 and 1:3 ratio or none, for 24h. ADSC enhanced rnCM in 1:1 ratio and HL-1 cells proliferation in both ratios (1 **F**, **H**). Representative micrographs of BrdUrd fluorescent staining (**A**, **C**) and phase contrast (**D**) are shown. Proliferation was assessed by immunofluorescent detection of 6h incorporated BrdUrd and quantification with Tissue Gnostics Tissue FAXS equipment. **B**) CM-DiI (red) labeled rnCM were co-cultured in absence, 1:1 and 1:3 ratio of CFDA SE (green) labeled ADSC. Increase densities of rnCM were detected in 1:1 ratio co-culture with ADSC (p < 0.05 1) **G**) dTomato lentivirally-tagged HL-1 cardiomyocytes were co-cultured in absence, 1:1, 1:2, 1:3 or 1:4 excess of eGFP lentivirally-tagged ADSC. Increased densities of dTomato HL-1 cardiomyocytes were detected after co-culture with eGFP positive ADSC in all ratios (p < 0.05, 1 **I**). **E**) Representative immunofluorescent micrographs of dTomato HL-1 cells in co-culture with eGFP ADSC are shown. Cardiomyocyte density was assessed by immunofluorescence detection of dTomato positive HL-1 cells and quantification with Tissue Gnostics Tissue FAXS equipment. Graphs represent triplicates (with SEM) data from n = 3 independent experiments from 3 donors.

We determined whether ADSC enhance the rate of cardiomyocyte proliferation in direct co-culture. In a 1:1 ratio, mitomycin-C-treated ADSC enhanced proliferation rate of rnCM 1.4-fold compared rnCM cultures alone (p < 0.05, Figure 1A, F). Higher ratios (1:3) of ADSC had no significant benefit (p < 0.05, Figure 1A, F). At the 1:1 ratio, the rnCM density increased 2.5-fold, yet at 3-fold excess of ADSC increases of rnCM were minimal (p < 0.05, Figure 1B, G).

As preparations of neonatal cardiomyocytes comprise are heterogeneous, we also assessed our findings with rnCM in the murine cardiomyocyte cell line HL-1. The proliferation rate of HL-1 cardiomyocytes was dramatically reduced by serum starvation and served to assess changes in the rate of proliferation by ADSC. HL-1 cardiomyocytes were co-cultured with ADSC in ratios 1:1 to 1:4. ADSC were pre-treated with mitomycin-C to induce cell cycle arrest. This allowed for the quantification of BrdUrd incorporation in actively proliferating HL-1 cardiomyocytes. ADSC significantly enhanced the rate of proliferation of HL-1 cardiomyocytes by 45% and 46% in 1:1 and 1:3 ratios compared to HL-1 cardiomyocyte alone (p < 0.05, Figure 1C, H). To investigate if various ratios of ADSC influence cardiomyocyte density, lentivirally eGFP-tagged ADSC (green) were co-cultured with lentivirally dTomato-tagged HL-1 cardiomyocytes (red). The HL-1 cell density doubled in a 1:1 and 1:2 ratio and further increased in a 1:3 and 1:4 ratio compared to HL-1 cardiomyocytes alone (p < 0.05, Figure 1B, D). ADSC enhanced HL-1 cardiomyocyte proliferation rate in all ratios, no significant differences were found between various ratios of ADSC to HL-1 cardiomyocytes (p < 0.05, Figure 1E, I).

### Conditioned medium of ADSC promotes the rate of proliferation of HL-1 cardiomyocytes

**Figure 2:**
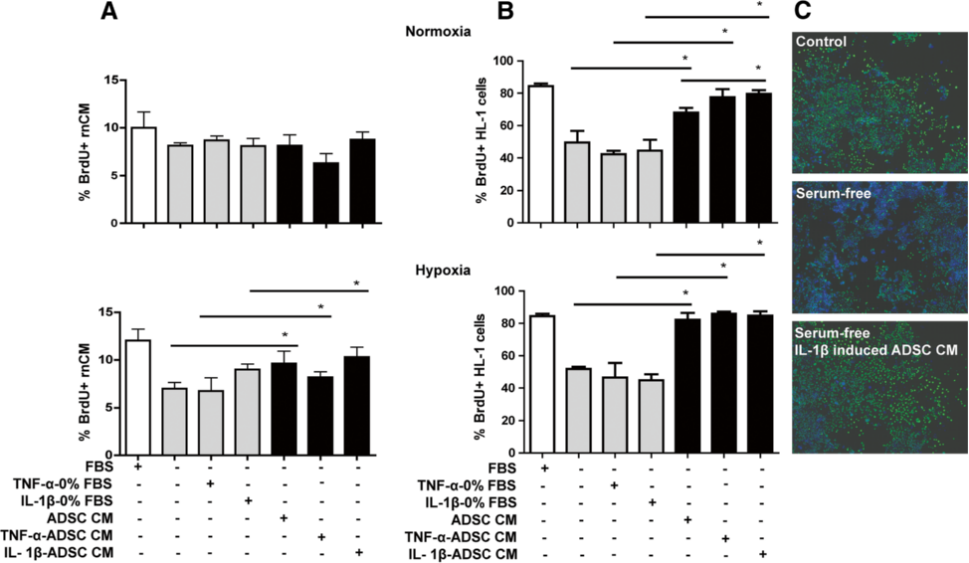
**Conditioned medium of ADSC promotes proliferation rate of cardiomyocytes.** Serum-free culturing reduced the proliferation rate of rat neonatal cardiomyocytes (**A**) and HL-1 murine cardiomyocytes (**B**) both under normoxic (21% oxygen) and hypoxic (2% oxygen) conditions compared to the serum control (10% FBS). This proliferation rate was not significantly influenced by treatment with TNF-α or IL-1β. Incubation of rnCM and HL-1 cardiomyocytes for 24h with serum-free conditioned media of human ADSC (n = 3 donors) pre-stimulated with TNF-α, IL-1β or none, restored the proliferation of HL-1 cardiomyocytes (p < 0.05). The stimulatory capacity of hypoxically preconditioned ADSC medium on the proliferation rate of rnCM and HL-1 cardiomyocytes under hypoxia was significantly larger than under normoxic conditions (p < 0.05). Proliferation was assessed by immunofluorescence detection of 6h incorporated BrdUrd and quantification with Tissue Gnostics Tissue FAXS equipment. **C**) Representative micrographs of immunofluorescence staining for BrdUrd of HL-1 cardiomyocytes in normal serum (10% FBS), serum-free or with serum-free ADSC conditioned medium after stimulation with IL-1β. Proliferation, *i*.*e*. BrdUrd incorporation – green, nuclei (DAPI) – blue. Graphs represent triplicates (with SEM) data from n = 3 independent experiments from 3 donors.

Possibly, secreted factors of ADSC could cause the enhanced proliferation rate of cardiomyocytes. The putative beneficial influence of conditioned media from ADSC was assessed on rnCM and HL-1 cardiomyocytes subjected to hypoxia (2% oxygen) and inflammation (TNF- α or IL-1β) (Figure 2A, B). In serum-containing media, approximately 10% and 12% of the rnCM proliferated respectively under normoxia and hypoxia. Serum starvation reduced the rate of proliferating rnCM to approximately 8% irrespectively of additional inflammatory factors (TNF-α or IL-1β) (p < 0.05, Figure 2A). Normoxic conditioned medium of ADSC did not change the rate of rnCM proliferation in high serum. Yet, after serum starvation the proliferation rate of rnCM increased 1.4-fold after treatment with normoxic conditioned medium of ADSC (p < 0.05, Figure 2A). The pre-conditioning of ADSC with TNF-α or IL-1β for the formation of the primed conditioned medium of ADSC resulted in respectively 1.2-fold increase in the proliferation rate of rnCM compared to TNF-α or IL-1β primed rnCM under hypoxia (p < 0.05, Figure 2A). To confirm the positive effect of the conditioned medium of ADSC on the enhancement of the cardiomyocyte proliferation rate, we performed the readout on the pure cardiomyocytes HL-1 cells.

In normal culture medium, approximately 85% of the HL-1 cardiomyocytes proliferated under both normoxic and hypoxic conditions. Serum starvation reduced the fraction of proliferating HL-1 cardiomyocytes almost two fold under normoxia or hypoxia. Treatment of serum-free HL-1 cardiomyocytes with TNF-α or IL-1β did not alter proliferation, irrespective of oxygen concentration. Serum-free-conditioned medium from normoxically cultured ADSC increased the proliferation rate of serum-free HL-1 cardiomyocytes by 18% compared to serum-free HL-1 cardiomyocytes control (p < 0.05). The proliferation rate of HL-1 cardiomyocytes under normoxic conditions was even further stimulated (35%, p < 0.05) upon incubation with conditioned medium from ADSC prestimulated with TNF-α or IL-1β compared to TNF-α or IL-1β stimulated serum-free HL-1 cardiomyocytes controls. The pro-inflammatory stimulation of ADSC with TNF-α or IL-1β to obtain primed ADSC conditioned medium ameliorated the cardiomyocyte proliferation rate as well. Furthermore, IL-1β primed conditioned medium of ADSC significantly increased (12%, p < 0.05) the HL-1 proliferation rate compared to nonstimulated conditioned medium of ADSC. Moreover, conditioned medium from hypoxia cultured ADSC ameliorated the proliferation rate of cardiomyocytes even further in comparison to conditioned medium of normoxia cultured ADSC (31% *vs*. 18% increase, p < 0.05). In addition, conditioned medium of hypoxia cultured ADSC in the presence of pre-stimulation with TNF-α or IL-1β showed only marginally improvement resulting in 40% increase in HL-1 cardiomyocyte proliferation rate (p < 0.05).

### Hypoxia and pro-inflammatory mediators upregulate IL-6 secretion by ADSC

**Figure 3:**
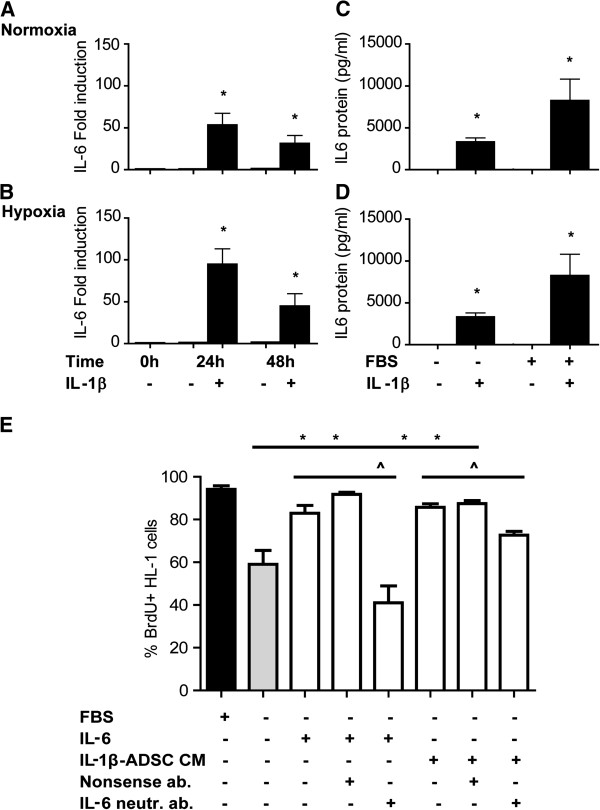
**Hypoxia and pro-inflammatory mediators upregulate IL-6 secretion by ADSC. A**, **B**) Quantitative RT-PCR analysis of gene transcript levels of IL-6 normalized to GAPDH expression. Gene transcript levels of IL-6 were strongly induced by stimulation with pro-inflammatory mediators (IL-1β) both under normoxia and hypoxia conditions within 24h and 48h (p < 0.05). The increase of IL-6 gene transcript level was far stronger after IL-1β stimulation (p < 0.05). **C**, **D**). Secreted IL-6, quantified by ELISA, was significantly increased after pre-stimulation of ADSC with the pro-inflammatory cytokine IL-1β (p < 0.05). Graphs represent triplicates (with SEM) data from n = 3 independent experiments from 3 donors. **E**, **F**) IL-6 is a positive regulator of cardiomyocyte proliferation. In serum-free medium, treatment with IL-6 restored the proliferation of rnCM and HL-1 murine cardiomyocytes compared to serum-free and serum (10% FBS) controls (p < 0.05). Similarly, pretreatment of human ADSC with IL-1β, yielded conditioned media that increase proliferation of rnCM and fully restored the proliferation of HL-1 cardiomyocytes in the absence of serum (p < 0.05). Addition of IL-6 neutralizing antibody to the IL-6 or conditioned medium of ADSC treated serum-free rnCM and HL-1 cardiomyocytes resulted in a significant decreased proliferation of rnCM and HL-1 cardiomyocyte (p < 0.05). Graphs represent triplicates (with SEM) data from n = 3 independent experiments, normalized to ADSC cultures before the treatment (0h).

Treatment of ADSC with IL-1β for 24 h or 48 h induced respectively 53-fold and 31-fold upregulation of IL-6 gene expression level (p < 0.05, Figure 3A).

Under hypoxia, treatment of ADSC with IL-1β for 24 h and 48 h resulted in higher increase of IL-6 gene expression, respectively by 95-fold and 45-fold ( p < 0.05, Figure 3B).

The level of secreted IL-6 showed a similar pattern as the gene expression after stimulation of ADSC with IL-1β (Figure 3C, D). Stimulation of ADSC with IL-1β , induced a 500-fold increase in IL-6 protein secretion within 24 h, which decreased to approximately 200-fold at 48 h both under normoxia and hypoxia (p < 0.05, Figure 3C, D).

### IL-6 secreted by ADSC enhances the cardiomyocyte proliferation rate

Stimulation with IL-6, increased the number of proliferating rnCM from 8% (serum free controls) to 9% (p < 0.05, Figure 3E). Addition of IL-6 neutralizing antibody to the IL-6 treated rnCM reduced the number of proliferating cells to 7% compared to IL-6 treated controls (p < 0.05, Figure 3E). Stimulation of rnCM with the serum free ADSC conditioned medium resulted in increase of proliferating rnCM to 8.5% compared to serum free controls. Addition of IL-6 neutralizing antibody to the conditioned medium of ADSC resulted in significant decrease of proliferating rnCM to 7.4% (p < 0.05, Figure 3E).

Adult HL-1 cardiomyocytes were cultured in the presence of 10% serum. Under serum-free conditions, IL-6 and the conditioned medium of ADSC induced a 24% and 27% upregulation of the proliferation rate of HL-1 cardiomyocytes respectively, compared to HL-1 serum-free control (p < 0.05, Figure 3F). Addition of IL-6 neutralizing antibodies to the IL-6 treated HL-1 cardiomyocytes reduced their proliferation rate by 42% (p < 0.05, Figure 3E) compared to IL-6 treated controls. Treatment of serum-free conditioned medium of ADSC with IL-6 neutralizing antibodies also reduced the rate of HL-1 cardiomyocyte proliferation rate by 13% compared to conditioned medium of ADSC (p < 0.05, Figure 3F).

### Conditioned medium of ADSC increase cell cycle progression gene expression profile in HL-1 cardiomyocytes

**Figure 4:**
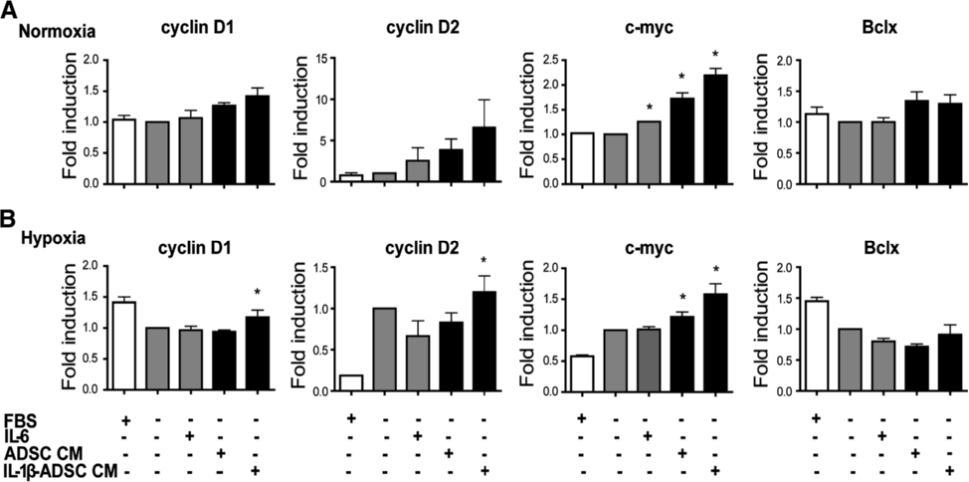
**Conditioned medium of ADSC augments the cell cycle progression gene expression profile of HL-1 cardiomyocytes. **Quantitative RT-PCR analysis of gene transcript levels of cyclin D1, cyclin D2 and c-Myc normalized to Beta-2 microglobulin expression. **A**) Gene transcript level of cyclin D1 and cyclin D2 did not change in HL-1 cardiomyocytes upon stimulation with IL-6 or ADSC conditioned medium in normoxia (p < 0.05). B) Gene expression of cyclin D2 showed a significant increase upon stimulation with IL-1β primed conditioned medium of ADSC under hypoxia (p < 0.05). **A**) Gene transcript levels of c-Myc were significantly upregulated upon stimulation with IL-6 or conditioned medium of ADSC and IL-1β primed ADSC conditioned medium under normoxia (p < 0.05). **B**) Under hypoxia gene transcript levels of c-myc in HL-1 cells showed significant upregulation upon stimulation with conditioned medium of ADSC and IL-1β primed conditioned medium of ADSC (p < 0.05). **A**, **B**) Gene transcript levels of apoptotic marker Bclx remained unchanged in HL-1 cardiomyocytes upon stimulation with IL-6 or conditioned medium of ADSC (p < 0.05). Graphs represent triplicates (with SEM) data from n = 3 independent experiments, normalized to serum-free HL-1 cardiomyocytes.

Cell cycle progression requires activation of cyclin complexes and G1/S phase transition and associates with increased expression of c-Myc, while anti-apoptotic genes such as Bclx are upregulated. Adult HL-1 cardiomyocytes were cultured in the presence of 10% serum. Serum-free HL-1 cardiomyocytes were cultured under normoxia and hypoxia in the presence of IL-6 or IL-1β primed conditioned medium of ADSC. Stimulation of HL-1 cardiomyocytes with the conditioned medium of ADSC from normoxia and IL-1β primed resulted in increased gene expression of cyclin D1 and cyclin D2 compared to serum-free HL-1 cells, yet not significant (p < 0.05, Figure 4A). Hypoxia and IL-1β primed conditioned medium from ADSC resulted in significant increase in the gene expression of cyclin D1 and cyclin D2 by respectively 1.2-fold and 1.3-fold increase compared to serum-free HL-1 cells (p < 0.05, Figure 4B). Stimulation of HL-1 cardiomyocytes with IL-6 both under normoxia and hypoxia did not significantly affected the gene expression of cyclin D1 and cyclin D2 (p < 0.05, Figure 4A, B). The elevated gene expression of cyclin D1 and cyclin D2, followed the significant increase of c-myc gene expression in HL-1 cells. Stimulation of HL-1 cells with conditioned medium of ADSC or IL-1β primed ADSC conditioned medium under normoxia resulted in significant increase of c-myc gene expression, respectively by 1.7-fold and 2.2-fold induction compared to control HL-1 cells (p < 0.05, Figure 4A). Stimulation of HL-1 cardiomyocytes with hypoxia and hypoxia with IL-1β primed conditioned medium from ADSC resulted in significant increase in gene expression of c-myc, respectively by 1.2-fold and 1.6-fold induction compared to control HL-1 cells (p < 0.05). Addition of IL-6 to HL-1 cells resulted in significant increase of c-myc gene expression only under normoxia by 1.3-fold compared to control HL-1 cells (p < 0.05, Figure 4A). IL-6 stimulation of HL-1 cells under hypoxia did not show significant change in HL-1 gene expression of c-myc compare to serum-free HL-1 cells (p < 0.05, Figure 4B). Stimulation of HL-1 cardiomyocytes with ADSC conditioned medium or IL-6 did not change expression of the antiapoptotic gene Bclx in HL-1 cardiomyocytes either under normoxia or hypoxia compared to control HL-1 cells (p < 0.05, Figure 4A, B).

### Conditioned medium of ADSC increases autocrine IL-6 gene expression in HL-1 cardiomyocytes

**Figure 5:**
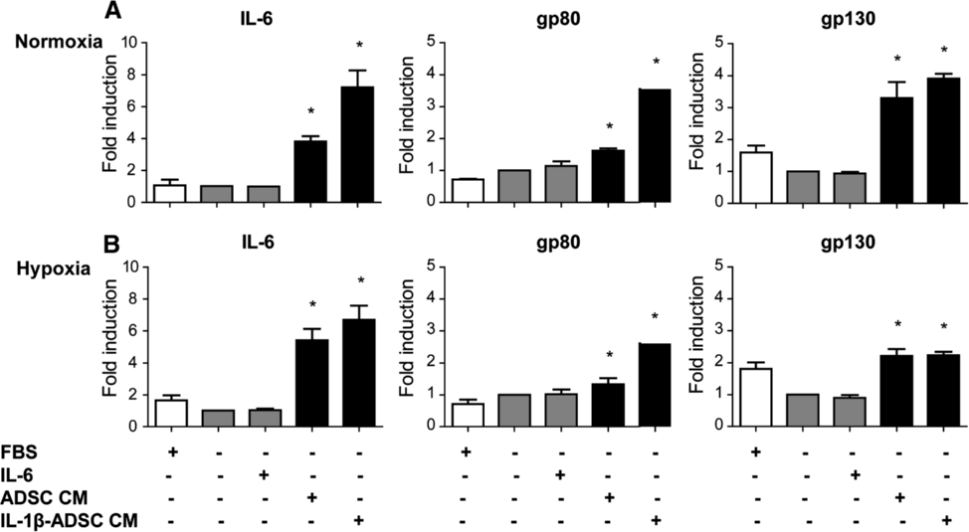
**Conditioned medium of ADSC increases autocrine IL-6 signaling gene expression in HL-1 cardiomyocytes. **Quantitative RT-PCR analysis of gene transcript levels of IL-6 and IL-6 receptor complex gp130/gp80 normalized to Beta-2 microglobulin expression. **A**, **B**) Stimulation of HL-1 cardiomyocytes with conditioned medium of ADSC and IL-1β primed ADSC conditioned medium resulted in a significant upregulation in gene expression of IL-6 and the IL-6 receptor components gp130 and gp80 (p < 0.05). Gene transcript level of IL-6 or gp130/gp80 did not change in HL-1 cardiomyocytes upon stimulation with IL-6 alone (p < 0.05). Graphs represent triplicates (with SEM) data from n = 3 independent experiments, normalized to serum-free HL-1 cardiomyocytes.

HL-1 cardiomyocytes were cultured in the absence of serum as a control. Stimulation of HL- cardiomyocytes with IL-6 under serum-free conditions did not effect the gene expression profile of IL-6, IL-6 receptor α (gp80) or IL-6 receptor β (gp130) both under normoxia and hypoxia compared to a serum-free control (p < 0.05, Figure 5A, B). Addition of ADSC conditioned medium to HL-1 cells significantly increased gene expression of IL-6 by 4-fold under normoxia and 5.4-fold under hypoxia compared to a serum-free control (p < 0.05, Figure 5A, B). Correspondingly, stimulation of HL-1 cardiomyocytes with conditioned medium of ADSC resulted in significant increase in gene expression of IL-6 receptor α (gp80) and β (gp130) by respectively 1.6 and 3.3-fold under normoxia compared to a serum-free control (p < 0.05, Figure 5A) and 1.3 and 2.2-fold under hypoxia compared to a serum-free control (p < 0.05, Figure 5B). Addition of IL-1β primed ADSC conditioned medium to HL-1 cardiomyocytes resulted in higher increase of IL-6 gene expression, resulting in 7-fold increase under normoxia and hypoxia compared to a serum-free control (p < 0.05, Figure 5A, B). Stimulation of HL-1 cardiomyocytes with IL-1β primed conditioned medium of ADSC resulted in significant increase in gene expression of IL-6 receptor α (gp80) and β (gp130) by respectively 3.5 and 3.9-fold under normoxia compared to a serum-free control (p < 0.05, Figure 5A) and 2.6 and 2.2-fold under hypoxia compared to a serum-free control (p < 0.05, Figure 5B).

### ADSC conditioned medium-dependent signaling pathways targeting the increased rate of cardiomyocyte proliferation

**Figure 6:**
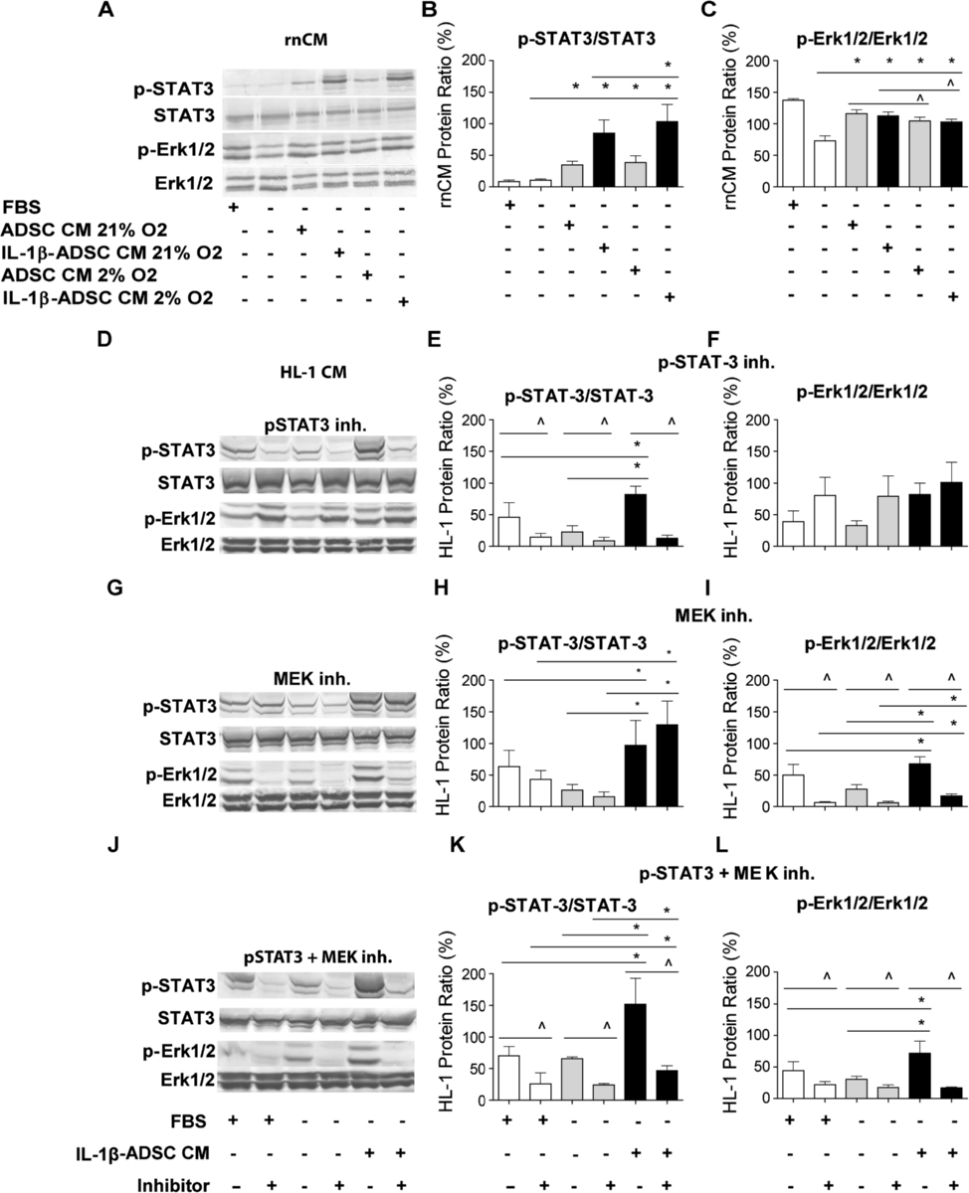
**ADSC conditioned medium-dependent signaling pathways targeting increase of HL-1 cardiomyocyte proliferation. **Representative examples of western blot analyses of phosphorylated STAT3 and phosphorylated Erk1/2 in **A**, **B**, **C**) rnCM stimulated with the conditioned medium of ADSC and IL-1β primed ADSC from normoxic and under hypoxic cultures. The stimulation of rnCM with the serum free conditioned medium of ADSC resulted in activation of STAT3 and Erk1/2. **D**, **E**, **F**) in HL-1 cardiomyocytes in the presence of STAT3 phosphorylation inhibitor (Stattic) **G**, **H**, **I**) MEK inhibitor (U0126) and **J**, **K**, **L**) both inhibitors (Stattic + U0126). Inhibition of STAT3 phosphorylation resulted in increased of Erk1/2 phosphorylation. Non-phosphorylated STAT3 and Erk1/2 levels did not change and indicate equivalent protein controls in all samples (n = 3).

The phosphorylation of STAT3 in rnCM did not depend on the presence of serum (Figure 6A, B). Yet, serum free conditioned medium of ADSC resulted in activated STAT3 by 4-fold both under normoxia and hypoxia conditions in serum starved rnCM (p < 0.05, Figure 6A, B). The peak of activation of p-STAT3 was reached in rnCM by stimulation with conditioned medium of ADSC primed with IL-1β both under normoxia and hypoxia resulting in respectively 8.5- and 10-fold increase compared to the serum free controls (p < 0.05, Figure 6A, B). In rnCM Erk1/2 was strongly phosphorylated in the presence of serum (p < 0.05, Figure 6A, C). Under serum free condition the phosphorylation of Erk1/2 was 2-fold decreased compared to serum control (p < 0.05, Figure 6A, C). The stimulation of rnCM with the serum free conditioned medium of ADSC and the IL-1β primed conditioned medium of ADSC resulted in the strong activation of Erk1/2, reaching 1.5-fold increase in compare to serum free controls (p < 0.05, Figure 6A, C) both under normoxia and hypoxia. The activation of Erk1/2 in rnCM by the serum free conditioned medium of ADSC was comparable to the level of phosphorylation in the rnCM stimulated with serum (Figure 6A, C). In HL-1 cardiomyocytes, STAT3 and Erk1/2 were both phosphorylated in the presence of serum. After 24h of serum deprivation, the phosphorylation *i*.*e*. activation, of these transcription factors was only slightly reduced (Figure 6D-L). The phosphorylation of STAT3 was decreased by 3-fold in the presence of the p-STAT3 inhibitor (Stattic) (p < 0.05, Figure 6D, E), while Erk1/2 phosphorylation was reduced by 8- and 4-fold with the MEK inhibitor (U0126) respectively in compare to the serum and serum-free controls (p < 0.05, Figure 6G, I). Remarkably, stimulation of HL-1 cardiomyocytes with serum-free IL-1β-stimulated-ADSC-conditioned medium resulted in a 2-fold increase in phosphorylation of STAT3 and Erk1/2, that reached higher level than serum controls (p < 0.05, Figure 6D-L). Blocking of STAT3 phosphorylation resulted in reduced levels of phosphorylated STAT3 and 2-fold increased phosphorylation of Erk1/2 (Figure 6D, F). In contrast, activation of phosphorylated STAT3 did not depend on activation of Erk1/2 phosphorylation (Figure 6D-L). Simultaneous inhibition of JAK/STAT and MAPK signaling pathway resulted in reduced levels of phosphorylated STAT3 by 2.7-fold and phosphorylated Erk1/2 by 2-fold (p < 0.05, Figure 6J-L).

### ADSC-dependent signaling pathways targeting HL-1 cardiomyocyte proliferation rate

**Figure 7:**
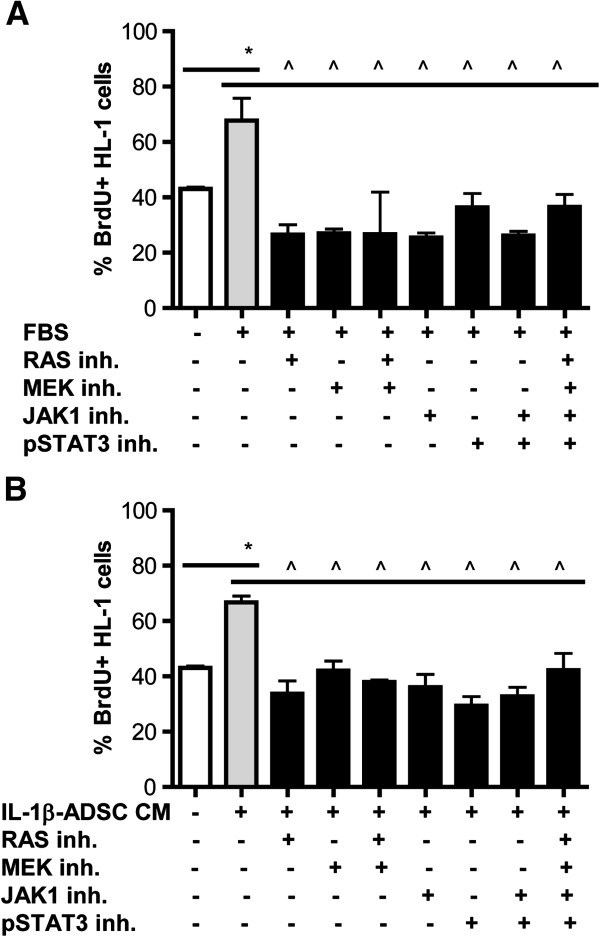
**ADSC-dependent signaling pathways targeting HL-1 cardiomyocyte proliferation. A**) Treatment of serum-free cultured HL-1 cardiomyocytes with FBS or **B) **serum-free conditioned medium of IL-1β stimulated ADSC resulted in enhanced proliferation of HL-1 cardiomyocytes (p < 0.05). **A**, **B**) Addition of inhibitors of upstream and downstream of MAPK (RAS and MEK) or JAK/STAT (JAK1 and pSTAT3) inhibitors all resulted in a significant decrease of proliferation of HL-1 cardiomyocytes (p < 0.05). Graphs represent triplicates (with SEM) data from n = 3 independent experiments.

In the presence of mitogenic factors such as serum and conditioned medium of ADSC, HL-1 cardiomyocytes showed an increase in proliferation (25% and 24% respectively, p < 0.05, Figure 7A, B). In the presence of serum addition of inhibitors targeting upstream or downstream of JAK/STAT (JAK1 and STAT3) and MAPK (MEK1) signaling pathway resulted in a decreased proliferation rate of HL-1 cardiomyocytes ranging from 31 to 41% (p < 0.05, Figure 7A). Pre-treatment of HL-1 cardiomyocytes with these inhibitors also reduced the mitogenic effect of conditioned medium of ADSC, observed as a significant decrease in the fraction of BrdUrd positive cells by 24 to 37% (p < 0.05, Figure 7B).

## Discussion

In this study we show that Adipose Derived Stromal Cells (ADSC) enhance the proliferation rate of both primary CM and a CM cell line, in a paracrine manner and in direct co-culture *in vitro*. One of the main stimulators secreted by ADSC was IL-6. The *in vitro* hypoxic and pro-inflammatory preconditioning of ADSC *i*.*e*. mimicking the post-myocardial infarction microenvironment (ischemia and inflammation), strongly upregulated the IL-6 production by ADSC and further augmented the stimulation of the proliferation of cardiomyocytes. The IL-6 stimulated cardiomyocyte proliferation was accomplished through activation of both Janus Kinase-Signal Transducer and Activator of Transcription (JAK/STAT) and Mitogen-Activated Protein (MAP) kinases (MAPK) mitogenic signaling pathways.

Stimulation of rat neonatal cardiomyocytes or HL-1 cardiomyocytes with conditioned medium of ADSC increased their proliferation rate. To mimic the behavior of therapeutic cells in the post-infarct cardiac microenvironment, we stimulated ADSC with hypoxia and pro-inflammatory mediators, which increased their production of IL-6. Remarkably, Efimenko and co-workers, showed that stimulation of MSC from bone marrow or adipose tissue with high concentrations of TNF-α did not alter their profile of pro-angiogenic mediators, which paradoxes to our finding that pro-inflammatory stimulation augmented regenerative potential of therapeutic cells [14]. The differences may be, that different stimuli (TNF versus IL-1) were used and different readouts, *i*.*e*. angiogenesis versus cardiomyocyte proliferation. Furthermore, our data indicate that hypoxia alone, but in particular together with a pro-inflammatory stimulus, augment CM proliferation by ADSC conditioned media too. This indicates that hypoxia can further augment the regenerative potential of ADSC. In contrast to current data, not only hypoxia may exert a beneficial effect on ADSC [15,23]. We found that inflammation had far stronger effect on the ADSC secretion profile. Although hypoxia itself did not alter IL-6 gene expression levels by ADSC, in combination with inflammatory mediators enhanced regenerative potential of ADSC.

Stimulation of rnCM and adult HL-1 cardiomyocytes with IL-6 resulted in an enhanced level of the cardiomyocyte proliferation rate. Targeting IL-6 with neutralizing antibodies against IL-6 in the presence of IL-6 or conditioned medium of ADSC resulted in decreased rate of cardiomyocyte proliferation. The blocking of IL-6 in ADSC conditioned medium only partially inhibited positive effect of ADSC conditioned medium on cardiomyocyte proliferation rate. This suggests that either conditioned medium of ADSC contains additional only mitogenic factors or that other factors promote rnCM and HL-1 cardiomyocyte proliferation rate synergistically with IL-6.

This is corroborated by our observation that stimulation of HL-1 cardiomyocytes with conditioned medium of ADSC resulted in a significant increase of c-myc and IL-6 receptor complex gp130/gp80, while stimulation with IL-6 alone did not show significance gene expressions changes.

**Figure 8:**
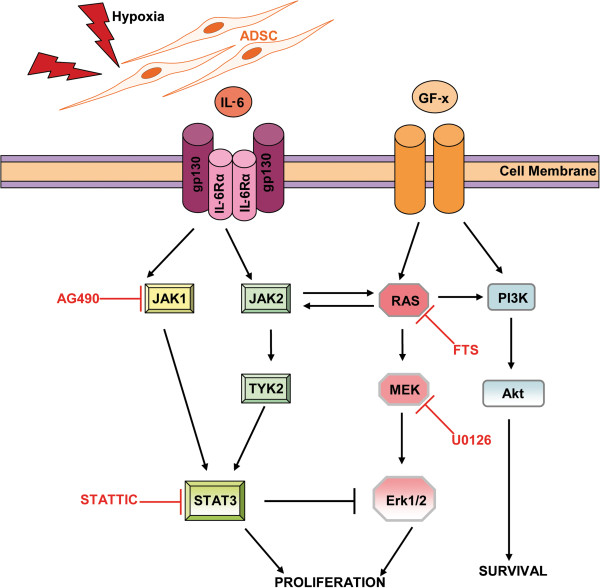
**Schematic representation of proposed Adipose Stromal Cell-derived IL-6 signaling pathway.** The ADSC-secreted cytokine IL-6 and growth factors, which enhance the proliferation of cardiomyocytes through canonical signaling *i*.*e*. STAT3 activation and through non-canonical signaling *i*.*e*. Erk1/2 activation. The balance between STAT3 and Erk1/2 signaling is governed by STAT3, which can suppress Erk1/2 activation, through an unknown intermediate. In the absence of STAT3 signaling, IL-6-RAS-activated Erk1/2 can compensate for the potential loss of stimulatory effect on the proliferation on cardiomyocytes.

IL-6 signaling involves activation of downstream signaling of two major signaling pathways *i*.*e*. JAK/STAT and MAPK-Erk1/2 that are mitogenic in various cell types. Thus, we analyzed the significance of both of these pathways on cardiomyocyte proliferation rate. Our study identifies a previously uncharacterized function of conditioned medium of ADSC signaling in regulating cardiomyocyte proliferation. Stimulation of rnCM and HL-1 cardiomyocytes with conditioned medium of hypoxically and proinflammatory primed ADSC resulted in strong phosphorylation of STAT3 and Erk1/2, the downstream targets of JAK/STAT and MAPK activation (Figure 8).

Similarly, previous studies on skeletal muscle have shown that regular exercise causes damage that is followed by increased IL-6 level. The released IL-6 activates the JAK/STAT signaling pathway and augments repair of skeletal muscle [24]. Recent clinical therapies with postconditioning of the ischemic heart show beneficial effect on the reduction of the scar size due to the activation of STAT3 [25] and involvement of IL-6 in this process [26]. In addition, pro-inflammatory cytokines such as TNF-α related TWEAK or ligands from EGF family such as neuregulin and HB-EGF provided evidence for engagement MAPK in induction of the cardiomyocyte proliferation rate [27-29].

Conditioned medium of ADSC activated the downstream JAK1 and JAK2/TYK2 that lead to their target STAT3 Tyr^705^ phosphorylation in rnCM and HL-1 cardiomyocytes. Blocking of JAK1 with commonly used JAK/STAT inhibitor did not diminished the level of phosphorylated STAT3, suggesting that JAK/STAT activation can also occur through JAK2/TYK2 [30]. Remarkably, direct inhibition of phosphorylated STAT3 with Stattic resulted in reduced STAT3 and increased levels of phosphorylated Erk1/2 (Figure 6). This suggests that the stimulated proliferation rate of HL-1 cardiomyocytes is a balance between STAT3 signaling and MAPkinase signaling [31]. Although prolonged inhibition of one of the upstream or downstream of JAK/STAT or MAPK pathways lead to decreased proliferation rate of HL-1 cardiomyocytes either in the presence of mitogenic factors or conditioned medium of ADSC.

The therapeutic benefit of stem cells for cardiac therapy is well-accepted, however the stem cell response to the host’s post-MI microenvironment is uncertain [32]. The main mode of action of cardiac stem cell therapy is through paracrine mechanisms. Indeed, the intravenous administration of conditioned culture media from bone marrow derived MSC in pigs improved cardiac remodeling and perfusion [33,34]. To unravel the mechanism of paracrine therapeutic benefit of cardiac stem cell therapy, we subjected cardiomyocytes to the conditioned medium of ADSC.

## Conclusions

The post-infarct cardiac microenvironment consists of an imbalanced level of inflammatory and anti-inflammatory mediators that correlate with the outcome of diseased myocardium. Cytokines might exert different function in time and dose dependent manner. Prolonged chronic high levels of IL-6 after MI are considered as a cause of hypertrophy and heart failure. Recent studies demonstrate that pro-inflammatory cytokines can activate cardioprotective signaling pathways in the post-infarct heart. IL-6 though might exert dynamic actions and act as a potent “myokine”, where in a rapid response to acute myocardial infarction it activates cardioprotective pathways [35], resulting in increase in cardiomyocyte proliferation. Application of the conditioned medium derived from therapeutic cells rather than cells themselves would circumvent the problem of retention in cardiac stem cell therapy. Additionally, the current approach of use of primed conditioned medium of therapeutic stem cells offer “off -the-shelf” product, which may be used for multiple injections.

## Competing interests

The authors report that there is no conflict of interest associated with this paper.

## Authors’ contributions

EP carried out the experimental studies, drafted the graphs, performed the statistical analysis and wrote the paper. GK have been involved in the design of the study, drafting the manuscript and revising it critically. MGLB have been involved in the molecular genetic studies. MCH have been involved in revising critically the manuscript and have given final approval of the version to be published. All authors read and approved the final manuscript.
